# Many knowledges for One Ocean

**DOI:** 10.1038/s44183-026-00231-z

**Published:** 2026-09-03

**Authors:** Elizabeth Havice, Noella J. Gray, Oscar Hartman Davies, Nadège Legroux, Jessica Lehman, Emily C. Melvin, Kimberley Peters, Teriitutea Quesnot, Alice Vadrot, Lisa Campbell

**Affiliations:** 1 Department of Geography & Environment, University of North Carolina-Chapel Hill, Chapel Hill, NC USA; 2 Department of Geography, Environment and Geomatics, University of Guelph, Guelph, ON Canada; 3 Division of History of Science, Technology and Environment, KTH Royal Institute of Technology, Stockholm, Sweden; 4 Helmholtz Institute for Functional Marine Biodiversity at the University of Oldenburg, Oldenburg, Germany; 5 Alfred Wegner Institute, Helmholtz Centre for Polar and Marine Research, Bremerhaven, Germany; 6 Department of Geography, Durham University, Durham, UK; 7 Nicholas School of the Environment, Duke University Marine Lab, Beaufort, NC USA; 8 Carl von Ossietzky Universitaet Mathematik und Naturwissenschaften, Institute for the Chemistry and Biology of the Marine Environment (ICBM), at Oldenburg, Oldenburg, Germany; 9 Department of Geography, Université Côte d’Azur, Nice, France; 10 Department of Political Science, University of Vienna, Vienna, Austria

**Keywords:** Climate sciences, Ocean sciences, Scientific community

## Abstract

The international ocean community gathered in Nice, France, in June 2025 under a *One Ocean* framing that unified participants around work for sustainable and equitable oceans. We explore ways that the *One Ocean Science* shared at the One Ocean Science Congress (OOSC) – including new data technologies promising ‘complete’ ocean renderings – makes space for difference in ocean knowledges and ways of knowing. Drawing on our work on new data technologies, oceans governance, and our participant observation at OOSC and the Third United Nations Oceans Conference, we explore three sites where universalism and difference were evident at the meetings: the propensity to render the oceans technical rather than political; the persistence of gaps as a defining feature of One Ocean Science; and sciences and knowledges incommensurable with One Ocean Science universalism. We highlight that One Ocean Science universalism is, perhaps counterintuitively, constituted *through* differences and heterogeneities in scientific practices and politics. Manifesting the United Nations Decade of Ocean Science for Sustainable Development’s goals of ocean science that fosters equity and sustainability will require holding space for difference within One Ocean, including by interrogating and redesigning the epistemological infrastructure of One Ocean Science so that pluralism becomes structural rather than residual.

## Introduction

In June 2025, over 2000 scientists gathered in Nice, France, for the One Ocean Science Congress (OOSC). The meeting was scheduled the week prior to, and co-located with, the Third United Nations Ocean Conference (UNOC3), a gathering of state and non-state actors to take stock of and promote progress towards UN Sustainable Development Goal 14 - Life Below Water (SDG14). With an aim to “…provide Heads of State and government and society at large with a comprehensive understanding of the health and future trajectory of the ocean”, OOSC was an explicit attempt to advocate for “[s]cience-informed decisions [to] enable the conservation and sustainable use of the Ocean, optimize the solutions it offers, and safeguard the services and benefits it provides to humanity” (see: https://one-ocean-science-2025.org/home.html). To facilitate scientific input to UNOC3, OOSC’s program and its *Recommendations to Heads of State and Government*^1^ adopted a structure that matched that of UNOC3 proceedings, and both OOSC and its Recommendations were mentioned multiple times during UNOC3. According to UN Ambassador for the Ocean, Peter Thompson, OOSC was a new model for scientists wishing to influence governance via “science diplomacy” (UNOC3 Side Event 5, 9 June 2025).

The *One Ocean* in the Congress’s name reflects a frequent framing of ocean-related diplomacy and high-level events. Scientists, philanthropists, non-governmental and multilateral organizations now commonly invoke One Ocean, a result of coordinated efforts by ocean literacy advocates, oceanographers and marine scientists (including marine social scientists) to recast multiple, geographically distinguished oceans – e.g. the Pacific Ocean, Atlantic Ocean – as a single planetary entity connected by, for example, global circulation patterns. Reference to One Ocean contrasts with only a decade ago, when the language of oceans (plural) was articulated in SDG14. Today, the UNESCO Intergovernmental Oceanographic Commission (IOC-UNESCO)’s Ocean Literacy Campaign’s first principle is that: “The Earth has one big ocean with many features” (see: https://oceanliteracy.unesco.org/about). Other principles emphasize the ocean’s size and importance, i.e., that the ocean covers 70% of the Earth’s surface and large-scale oceanographic processes function at the planetary scale (e.g., the Coriolis effect arising from the Earth’s rotation). One Ocean contains 97% of Earth’s water and has absorbed 30% of total anthropogenic carbon dioxide emissions^2^, making it integral to global water and climate systems.

While less than 20% of One Ocean has been explored (an oft-cited and contested statistic at OOSC and UNOC3), the IOC Ocean Literacy Campaign notes that technologies such as satellites, drifters, buoys, mathematical models and interdisciplinary collaboration will expand scientific understanding of One Ocean. In the context of the ocean data revolution, technologies and tools from satellites to submersibles to the infrastructures developed to collate, analyze, and make sense of ocean data, aim to provide global and complete renderings of One Ocean. That is, One Ocean is now imaginable and seemingly knowable via (new) data technologies, exemplified by flagship programs for ocean observation, like the Global Ocean Observing System (GOOS) coordinated by IOC-UNESCO^3^ as well as the range of ocean data science initiatives aiming to mobilize data and technology for ocean improvements^4^. (New) data technologies refer to methods of data collection and generation, data themselves, and the platforms, analytical techniques, and infrastructures developed to make sense of them (not all of which are new), at times so they can be applied for management purposes^5^. With more data now generated about the ocean in a year than over the past century^6^, a new era of ocean discovery promises improved ocean governance^7^.

**Fig. 1:**
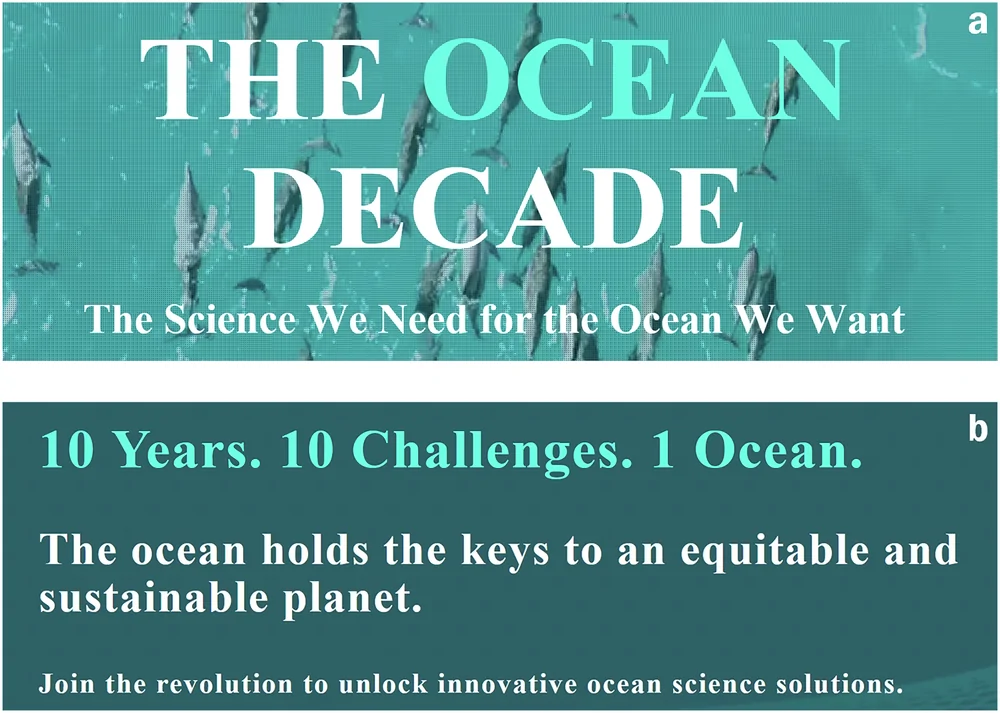
The United Nations Decade of Ocean Science for Sustainable Development. **a** The Decade’s tagline emphasizes science for a single ocean that a unified, but unspecified, ‘we’ want. **b** Equitability and sustainability are key principles of the Decade’s work for One Ocean. Images are screen captures from http://oceandecade.org, accessed August 2025.

These characteristics of One Ocean suggest a need for science ‘at scale’: One Ocean *Science*. The United Nations Decade of Ocean Science for Sustainable Development (2021–2030) (from here, the Ocean Decade or Decade) further links One Ocean and One Ocean Science. The Ocean Decade, also coordinated by IOC-UNESCO, provides a convening framework for “a wide range of stakeholders across the world to engage and collaborate outside their traditional communities to trigger nothing less than a revolution in ocean science” (see: https://oceandecade.org/vision-mission/). While there have long been efforts to study the ocean at a planetary scale, the Decade offers a new kind of opening for this work. It aims to convene coordinated scientific projects to create One Ocean Science under a theory of change that doing so will unlock ocean science solutions. Solutions are to support the desired outcomes of the Decade: equity and sustainability in One Ocean and the practices of knowing One Ocean to the benefit of the planet and all on it. Indeed, the Ocean Decade’s tagline is that it will provide the science we need for the ocean we want, though the scope of 'the science' and who that ‘we’ is in its global homogeneity, are left underspecified (Fig. 1a, b)^8–10^.

One Ocean Science is an articulation – and a practice – of the One Ocean concept. In this context, as we participated in OOSC, we were somewhat surprised that presentations, sessions, and keynotes reflected not a singular understanding of ocean spaces or processes, nor agreement on the possibility of complete knowledge offered via new data technologies that one might expect of a One Ocean Science that is working towards a vision of *One* Ocean. Rather, a diversity of trends in thinking about and studying oceans were present, including tensions between the ocean as technical or political; the presence of gaps within One Ocean Science; and the challenges of incommensurable knowledge types in knowing oceans. These observations, or openings, inform our analysis to follow. We explore attempts to balance the universalism of One Ocean with the diversity of many oceans and many approaches to knowing them. That is, we aim to contribute to a discussion that draws on our collective work on new data technologies and oceans governance as well as reflections on our participant observations at OOSC and UNOC3, to highlight that the universalism of One Ocean Science is, perhaps counterintuitively, constituted *through* differences and heterogeneities in scientific practice and politics.

## Results

### Universalism and heterogeneity in One Ocean Science

One Ocean and its link to One Ocean Science is a form of universalizing: it centralizes attention to the marine environment to overcome the relative lack of scientific and policy attention to this part of the planet, and to express the urgency of doing so in the context of accelerating and spatially extensive environmental change. One Ocean is universal in the way that it signifies connectivity, common challenges, and the need for global understanding of the ocean to inform management and cooperation therein. Scientific universalism is a key part of this work; as Somsen (2008, pg. 362) puts it, scientific universalism is “the idea that science is independent of the place where it is practiced (because of the universality of its knowledge or method), and that scientific practitioners are automatically united in a single global pursuit – a model of peaceful cooperation.”^11^

Universalism in oceanic thinking is not new. In Western thought, the idea of a singular ocean predates current articulations of One Ocean, and emerged out of a specific set of sciences and images. The travels and writings of Alexander von Humboldt were particularly influential^12^. In recent decades, One Ocean and One Ocean Science universalism builds from Earth Systems Sciences’ understanding of the Earth as one integrated system and, within it, the “world ocean” as “one dynamic entity with a special relationship to life on Earth”^3^.

Yet, this longstanding universalist vision of the oceans and ocean sciences is full of tensions because making universal, or ‘generalizing’^13^, sits uneasily with differences within scientific practice as well as among place-based knowledge systems and relationships^14^. Oceanic universalism is in tension with the heterogeneity of oceans and the experiences with data, technology and knowledge (and knowledge gaps) generated through scientific practice^15^^,^^16^. It also risks erasing the uneven power dynamics that have shaped the practice of ocean science and that dominate thinking about paths forward. As Jessica Lehman writes^17^, universalising strategies (such as the Anthropocene concept) “elide difference, inequality, and contingency in positing a unitary human species”^18,19^. In pointing to such tensions in an ocean context, Sammler and Peters^8^ ask: who is the singular ‘we’ of the UN Ocean Decade?

Such questions were not ignored at OOSC. Within one conference (OOSC), about One Ocean, acknowledgements of the multiplicity of ocean knowledge production were common. For instance, one speaker described the “global ocean” as “far from being a self-defined category” but one “contested by different actors (…) not a view from above or nowhere (…) but a view from some groups of scientists with specific sources of funding” and instruments (OOSC, Ocean observation governance in the 21st century: What history tells us about the future, 4 June 2025). In other words, the One Ocean drive of the One Ocean conference was simultaneously being challenged from within.

We join this reflection to urge attention towards “competing notions of what the ocean is, or what oceans are” which in turn inform discussion of how they are “used, deployed, coordinated and managed within a context of oceanic and planetary crisis/es”^8^. We emphasize the need for attending to differences in the ways of knowing and studying oceans, particularly as some new data technologies promise complete ocean renderings. From here, we explore the three aforementioned openings around the ways that One Ocean Science in the digital era is constituted through, and enriched by, difference. These arenas are not mutually exclusive or well bounded; dynamics bleed across and inform each other.

### Rendering technical

One Ocean Science turns attention to the scope of scientific work that is needed ‘for the ocean we want’, and, for some, new data technologies make the ideal of full coverage both an aspiration and a technical possibility. This includes the technical elements of data collection, storage, sharing, and dissemination, and these efforts unify much of the ocean science community around delivering enhanced renderings of the ocean. Yet, rendering the oceans is more than a technical project, and the work of making such technical advancements can obscure the political, cultural and social dimensions.

Indeed, social scientists examining scientific exercises that define problems and subsequent policy actions urge attention to the ways that scientific work can make problems appear technical, rather than political and cultural in nature^20^, and can occlude debates, doubts, and uncertainty^21^. This is what Tania Li^22^ calls “rendering technical”, a shorthand for a set of practices concerned with representing “the domain to be governed as an intelligible field with specifiable limits and particular characteristics… defining boundaries, rendering what lies within them visible, assembling information about that which is included and devising techniques to mobilize the forces and entities thus revealed”^23^. These dynamics also exist in the oceans. For instance, according to Lehman^24^ with regards to ocean observations, “[t]he issues raised by new ocean observing practices and accompanying technologies are not simply practical, involving the allotment of research dollars and the focus of capacity building efforts. They also pertain to core issues regarding the definition of socially relevant ocean science as well as geopolitical inequalities and tensions.”

There is a politics of knowing the oceans, and these emerge within the political history of data collection, ownership and use in ocean science^25^. Scholars of science and technology draw attention to the ways in which technical renderings of the environment, rather than producing ever-more-accurate pictures of reality, emerge from particular social and historical contexts^26^. Historically, work in oceanography – physical, chemical, biological, and geological – has been privileged in contemporary (Western) ocean science and governance^27–29^. Nation-states with strong maritime economies, histories, and multistate collaborations have led the capital and time intensive scientific work of knowing oceans (in particular the USA^30^). To date, scientists and institutions based in wealthy countries have played the primary role in defining research agendas, undertaking data collection and analysis, and training scientists^31^. There is a risk that, in emphasizing principles such as interoperability, open access, and complete coverage, One Ocean Science obscures the multiple contexts from which this knowledge emerges, as well as differing implicit values and priorities^32,33^.

Speakers at OOSC recognized and grappled with this. They noted that the technical project of knowing the oceans on one hand promises completeness (or at least increasingly multi-layered approximations), and on the other hand comes with risks of deepening a range of digital divides that can emerge in day-to-day scientific practices ranging from sites on vessels to labs to advanced observing systems to conference venues. ‘Capacity’ to participate in One Ocean Science is not just about contributing to a global scientific project, but about defining problems and ‘solutions.’ Speakers commonly asked: who does the science, and who is able to access and use the science? For instance, a researcher described a collaborative project to build “The Ocean Microbiome genetic resource: equitable access and shared capacity for the benefit of One Ocean, One Health”, a two-year vessel-based data collection project co-designed among Europeans, South Americans and Africans that includes attention to research methods for on-board data collection and subsequent analysis that offer scientific training and defines ‘best practices’ for sharing joint ownership of genetic resources and digital sequence information among all members of the consortium (OOSC, Tools, Data and Collaboration to support equitable access to marine genetic resources, capacity sharing and benefit sharing, 3 June 2025). Keynote Speaker Sheena Talma outlined her work to prioritize deep-sea research in the Indian Ocean, emphasizing that questions such as “who is the researcher?” guide her efforts to build research in the Seychelles that is done by Seychellois and that ensures trust between community and scientists (OOSC, Understanding prioritized deep-sea research in the Indian Ocean, 6 June 2026)^34^.

There is also the question of how technical renderings of the ocean will be used. Enthusiasm for technical processes that will provide complete renderings of oceans is based on a theory of change that more complete knowledge will lead to ‘better’ outcomes. In practice, technologies that lead to knowing the oceans reflect and drive certain dominant political and economic patterns, whether these are intentional or not. Ocean data science is itself an increasingly important economic sector of the ocean economy and the outputs of oceanic data collection and analysis are mobilized for political aims. For instance, as with GOOS, “[t]he geopolitics of the ocean is fundamentally based on these systems of observation and their ability to be recombined in ad-hoc ways to govern not only the present but also many possible futures through modeling and forecasting”^3^. Some speakers raised these questions, for instance, one UNOC3 speaker at a session on new Chinese technologies designed to inform decision making asked: “all high quality data are in well-funded institutions and regions, so how can we make sure the data can be shared by global citizens?” (UNOC3, Approaching intelligent and inspiring ocean, 9 June). While critical questions were present, the matter of who will actually do what with scientific renderings was seldom discussed at OOSC.

The technical and political are always entangled in efforts for rendering spaces and natures. There is a risk in the drive for One Ocean Science that the technical project masks the politics of knowing and representing the oceans^35^^,36^. As One Ocean Science works to answer the technical questions of how to collect, share, store, make data interoperable, and to explore the exciting technical possibilities therein, there is a need to think about the program as political and economic and cultural as well as technical. It was evident at OOSC and UNOC3 that many scientists are thinking along these lines because they aspire for One Ocean Science to inform the global sustainable development regime, and they recognize that it could easily replicate the colonial science of the past (OOSC, Plurality of value and knowledge systems, including indigenous and local, 3 June 2025). However, even when capacity-building efforts succeed in broadening access to ocean data technologies, the epistemological architecture embedded in those tools – their spatial primitives, their ontological assumptions, their models of what counts as valid data – remains largely unchanged, such that inclusion does not necessarily translate into epistemic equity. Awareness of the political must extend beyond a concern for access and capacity building (see (In)Commensurabilities, below). This is particularly important because the new blue economy is increasingly recognized as a digital knowledge and data economy (see e.g., https://www.noaa.gov/blue-economy).

### Gaps

Knowledge gaps about the oceans – and the need to fill them – are an animating force for One Ocean Science^37^. New data technologies are central in this effort because they offer possibilities for universal, automated and real time data collection that suggest the possibility of bypassing the political dimensions of ocean science noted above. One Ocean Science unites the scientific community around *full* data coverage as an imperative. Indeed, Vadrot and Wanneau^38^ note a paradigm shift among marine biodiversity monitoring communities at multiple epistemic sites including negotiating rooms, scientific fields, laboratories, and conferences emphasizing “the urgency of finding solutions to close data gaps and provide policymakers with readily available knowledge. Speeding up data collection—with the help of new devices, modelling, and artificial intelligence (AI) —was presented as the way forward to prevent further damage to the marine environment, a precondition being close cooperation between providers and users of data and technology.”

Those programs that promise complete coverage are high profile, but this vision also belies the always partial nature of ocean knowledge^39^ and the fact that all knowledge is situated^40^. Scientific data reflect legacies of social inequality^32,41^ and are constituted from uneven and incomplete data sources. As such, this vision of the ocean is at once contradicted by the very science rendering it, while also rendering the politics of data that so many scientists acknowledge, invisible (see Rendering Technical, above). Noting these tensions is vital for renderings of the ocean that often support the governance priorities of particular, powerful ocean actors^42^ contributing to selective world-building inherent to the field of marine biodiversity research where collaboration capital is unequally distributed^29,43^. These concerns also mirror longstanding questions about how data gaps reflect priorities in society as knowledge is bifurcated regionally (north/south) and scientifically (natural science/social science)^44^.

OOSC participants were aware of gaps, though in the sessions, the ambition for completeness was evident and there was a sense that technological tools will lead the way. For instance, in a keynote presentation, Takashi Gojobori stated, “Data makes our ocean one” (OOSC, Blue economy and smart ocean in sustainable developments, 4 June 2025). Yet the interplay between completeness and gaps is ever-present in One Ocean Science. For instance, the drive for a *complete* map of the seabed (e.g. via the Seabed 2030 Project) has generated momentum toward comprehensive, interoperable bathymetric data; simultaneously, presenters at OOSC highlighted the absence of ecologic, biologic and social (e.g. oral histories of the seabed) data in seabed mapping work, and pointed to the many potential consequences of such gaps (OOSC, Effective and equitable stewardship of the deep ocean, 3 June; OOSC, Human and non-human rights panel, 6 June)^35^.

Tensions between complete coverage and data gaps are also evident in the drive to create a Digital Twin of the Ocean (DTO), the focus of several OOSC panels. Presented as living, learning replicas of the marine world for improved ocean decision-making, Digital Twins underpin the Ocean Decade Challenge 8 to “create a comprehensive digital representation of the ocean”^45,46^. However, panelists acknowledged that any Digital Twin is only as powerful as the data and cooperation that underpin it, noting, for instance, that most DTO examples focus on shallow (above 200 m) and territorial waters in countries with the capacity to develop these computationally demanding systems. When prompted to reflect on knowledges and entities that will be difficult to digitize, panelists gave vague answers about the potential of AI to expand inclusion of local and Indigenous knowledges in the future, and acknowledged - in contradiction to the goal - that a ‘comprehensive’ global ocean Digital Twin is not currently possible (OOSC, Designing Digital Twins of the ocean, 4 June 2025).

One Ocean Science is simultaneously moving towards ‘the complete’ *and* comprised of gaps. Attention to the blank spaces and blind spots on the emerging maps and renderings of the oceans draws attention to the kinds of science and knowledges that fit (and do not) in One Ocean Science, and which are addressed or erased by technological advances. It raises questions about data coverage, completeness, and what will always remain unknown. There is also a critical question about how AI might fill those unknowns (and rendering ‘other’ knowledges less relevant) in its capacity for (big) data processing and advanced modelling. In one keynote session at OOSC focused on so-called ‘smart’ oceans, it was noted that AI has to be used “as much as possible” (OOSC, Blue economy and smart ocean in sustainable developments, 4 June 2025). Another session noted that there is a gap in asking what society needs data for so that new knowledge can be directed appropriately. Speakers noted, for example, that GOOS was not only a critical data infrastructure but a ‘societal’ one, with more knowledge required on complex societal needs (OOSC, Ocean observation governance in the 21st century: what history tells us about the future, 4 June 2025).

Attention to gaps keeps the many oceans of the One Ocean front of mind; it is not just where gaps exist but also the efforts to fill them that reveal the plurality and politics of ocean knowledge. Debates about the nature and distribution of knowledge gaps, the imperative of addressing them, and the mobilization of gaps to justify funding, research activity, and technological experimentation all reveal much about both the construction of One Ocean and its persistent fracturing. Indeed, while addressing some data gaps may lead to improved governance and advance justice claims, filling knowledge gaps is as political as neglecting them.

### (In)commensurabilities

Tensions within One Ocean Science are further reflected around issues of commensurability. Commensurability turns attention to the kinds of science and knowledge that do and do not fit into, and thus construct, One Ocean. Commensurability points to gaps in coverage noted in the section above, and specifically to the ways that techniques in ocean science can privilege some forms of knowledge (e.g. those that are able to be gathered with new data technologies) and overlook, erase and exclude others because of the difficulty of making them ‘fit’ into the scientific tools that make One Ocean.

Participants at OOSC recognized and grappled with the commensurability question, identifying a range of incompatibilities inherent in One Ocean Science and attending to methods for addressing them. For instance, keynote speaker Michelle Bender highlighted that understandings of the oceans as alive with histories, kinship, and understanding are often overlooked in ocean science and governance. Bender proposed a Rights of Nature approach that can recognize nature as the subject of legal rights guided by moral consideration, intrinsic values, and interconnectedness as a potentially “powerful but underutilized lever for change” in mobilizing ocean science. This presenter suggested that a Rights of Nature approach would result in a paradigm shift from ‘the science we need for the oceans we want’ to the science the *ocean* needs to ensure its vital processes and functions (OOSC, Ocean rights: Human and non-human rights to a healthy ocean, 4 June 2025). Some participants used the OOSC venue to make space for knowledge generation and exchange beyond the expert panel format that privileges Western science. For instance, OOSC organizers developed a scientific community event, *For the Ocean*, at the Nice Opera House. This event, which was also published on YouTube, used music, narrative, story-telling and visual imagery to communicate the high-level points in the manifesto that OOSC presented to UNOC3 leaders. Likewise, one OOSC Townhall experimented with format: rather than a panel of experts presenting knowledge (by far the dominant approach to knowledge sharing at OOSC), townhall organizers broke the attendees into groups (against universalism) and initiated small-group discussions including in non-English language clusters to gather participant perspectives, experiences and values about the oceans (OOSC, Bringing the outside, in: Voices for the science we need for the ocean we want, 4 June 2005).

Local and Indigenous knowledge was perhaps the most prominent incommensurability at OOSC. Mismatches between dominant knowledges and data collection systems and those held in local and Indigenous communities have long been points of contestation in ocean management^47–49^. Recent years have seen explicit leadership in attending to these tensions. For instance, in the Aha Honua declaration, Indigenous Peoples called on the global ocean observing community to recognize the knowledge of Indigenous peoples^50^, and examples of how to do this are beginning to emerge^51^. Rather than try to make Indigenous knowledge compatible or commensurable with dominant knowledges, such work examines how Indigenous knowledges can be digitized and coordinated with, or used alongside, Western science, specifically ocean observing systems. Considering distinct knowledge systems alongside one another, as equal, follows a two-eyed seeing approach, a concept introduced by Mi’kmaq Elder Albert Marshall^52^. However, digitizing Indigenous knowledge always holds danger of ontological violence - assimilating rather than upholding Indigenous ontologies^53–55^, and not all knowledge should be digitized or shared because of concerns related to data sensitivity, intellectual property, and cultural protocols, among others^51^. Scholars working on these issues around Indigenous knowledge specifically suggest that ultimately, “building meaningful, respectful, and reciprocal relationships with Indigenous communities is the only path forward for ocean observing systems”^51^. However, such relationships are context-specific, place-based, not universal and generalizable, respecting the data sovereignty and distinct knowledge systems of diverse Indigenous communities.

Throughout the meetings, participants frequently raised questions and probed the edges of the challenges and desirability of commensurability between Western and Indigenous knowledge. For example, Karen Fisher et al. questioned the very “kinds of science” produced under the umbrella of “data”. She noted Indigenous work is science, and the need for Indigenous knowledge to be “reclaimed”. It is not something to be “given” or “included”. Indigenous knowledges transcends scientific data categories (i.e. the biophysical). This talk also challenged the scaling up of One Ocean discourses and stressed the importance of “looking to the local” (OOSC, Plurality of value and knowledge systems, including Indigenous and local, 3 June 2025). Another talk in the session by Pieter Romer and Maia Hoeberechts argued for the need for “cultural observations” over longer time scales than modern data acquisition and urged that qualitative data was vital. In their discussion of the Ocean Networks Canada's Ocean 3.0 data portal they showed how Indigenous knowledge could be “integrated” into ocean monitoring. Notably, and perhaps unsurprisingly, in sessions on plural knowledges, then, perspectives were not necessarily united (i.e., on whether knowledge could or should be ‘integrated’, could or should be ‘reclaimed’), though there was a united front on ideas of data sovereignty and knowledge. This stresses the point in this paper - that difference is replete within broader One Ocean Science framings.

Incommensurabilities around Indigenous knowledge and new data technologies – particularly mapping and spatial representations – were also highlighted. A presentation that focused on methodological adaptations to GIS illustrated the possibilities and hard limits of technical commensurability in Indigenous contexts. In Moorea (French Polynesia), efforts to ‘integrate’ local fishers’ spatial knowledge into marine spatial planning revealed that standard zoning tools – sharped and fixed boundaries, categorical classifications – were systematically misaligned with how Pacific islanders describe and live with the sea^47^. At OOSC, Teriitutea Quesnot proposed that a fuzzy-graph-based GIS approach, built from relations, fishing narratives and hand-drawn sketches translated into probabilistic spatial layers, offered a more responsive technical format: one that preserves gradational, relational boundaries rather than imposing crisp lines. Yet even these more flexible formalizations function at best as boundary objects – bridging different knowledge systems together for planning purposes – rather than as genuine epistemic equivalents (OOSC, Inclusive science-policy-society interfaces, 5 June 2025).

Likewise, another presenter described a project related to the World Marine Protected Area (WMPA) Atlas. The WMPA aims to document all MPAs around the globe. Some communities argue that the WMPA encodes Western approaches to marking protected space to the exclusion of local and Indigenous ocean management practices. The presenter, Alexander Mawyer, highlighted a project in Hawai'i in which communities have created their own processes of documenting the overlaps between Indigenous-managed spaces and other jurisdictions. Part of the work is to ask if such efforts can counter the risk that the WMPA “gobbles up” and potentially displaces Indigenous knowledge of marine spaces and/or, alternatively, undermines sovereignty claims by operationalizing Indigenous concepts of ocean spaces within Western demarcation tools (OOSC, Human and non-Human rights, 6 June 2025).

Local and Indigenous perspectives and knowledges presented at OOSC are also not ‘complete’. For example, Pacific relations to the sea, which were frequently offered at OOSC, differ from Black oceanic relations in the Caribbean and the Americas, the latter of which are shaped by historical violences of the Atlantic slave trade^56,57^. Moreover, there is considerable diversity *within* regions, a point emphasized by a presenter who noted that diverse political circumstances and histories have led to divides across the Pacific with respect to deep sea mining (OOSC, One ocean: Many deep seas, 4 June, 2026). Incorporating Indigenous knowledge systems into Western systems risks homogenizing a plurality of knowledge systems, both across and within regions, points also addressed in a conference report^58^.

There is tension between the desire for commensurability and the technical imperatives of the One Ocean vision; between global, interoperable data and local, place-based knowledges and systems of recording. The distinction between One Ocean and many oceans rests between such universal and local concerns^59^. The point is to recognize the breadth, diverse knowledge and values shaping oceanic interactions^60^ and that some knowledges will not be commensurable with One Ocean Science. This was also evident in civil society activities within and beyond the official spaces of OOSC (and later UNOC3), where organisations (such as #SaveMaldives, Seas at Risk and the World Forum of Fisher Peoples) pointedly questioned the power dynamics at the heart of ocean solutions driven by dominant modes of ocean science (for example, highlighting science financing). The new data technologies ‘moment’ cannot ‘fix’ this incommensurability. So, how can the (One Ocean) Science community sit with such mismatches and differences among them? We note that sitting with incommensurability is not a passive position, it requires actively resisting the pressure to resolve difference, including through technical fixes.

## Discussion

The urgency and political momentum behind One Ocean Science is clear. Technical progress towards knowledge that renders One Ocean more visible and knowable is innovative, yielding advancements in understanding the oceans and informing goals from global to regional to local levels. To state the obvious, this progress is welcome as the need to know the oceans is crucial to efforts to sustain life on a warming earth. Yet engaging and questioning the politics of that knowledge is also vital.

The One Ocean framing, and its extension to One Ocean Science, aims to bring a particular ocean world, or ocean world-view, into being: a unified and singular ocean that ‘we’ – presumably the undifferentiated global community – want. The scientific community, including those at OOSC and UNOC3, grapple with the tension between using data and technology to make One Ocean, and recognizing the implications, exclusions and oversights that it entails. In One Ocean, many differences (and borders) remain between: spaces in the oceans; scientific disciplines and ways of knowing; North and South; OOSC and UNOC3 participants and non-participants, to name just a few.

We conclude by suggesting, following from Escobar^61^, that One Ocean can be, and perhaps already is, not a universal and singular process or project, but one that focuses on the interrelations *among* ocean worlds (plural), including “the conflicts that ensue as different ontologies strive to sustain their existence in their interaction with other worlds.” In drawing attention to differences within One Ocean Science, we signal the limits of One Ocean Science universalizing and urge attention to the conditions that allow for the flourishing of difference and heterogeneity therein. The summaries of presentations that we offer here, and the way that they are also captured in the OOSC manifesto, illustrate that we are not alone in this effort.

The work ahead involves critical engagement with the technical project of building a singular, legible and knowable ocean, and asking how One Ocean will be connected to defining the ‘ocean we want’. If the Decade’s goals of what ‘we’ want – an ocean science that fosters equity and sustainability – are to manifest via science and knowledge pathways, this involves holding space for difference within One Ocean that many in the One Ocean Science community are already pointing to. It involves recognizing that the technological process of making One Ocean has constraints and is not an even project around the globe. It involves exploring what One Ocean Science is accomplishing, what it can never accomplish, and what options it is precluding in defining its vision and practice. It involves asking if the counter currents within One Ocean Science are strong enough to make and sustain room for the diversity that comprises multiple oceans and ocean knowledges. This requires not only making room for difference within existing frameworks, but interrogating and redesigning the epistemological infrastructure of One Ocean Science itself – its data models, its spatial primitives, its standards of validity – so that pluralism becomes structural rather than residual.

## Methods

### Collaborative event ethnography

International conferences are places where stakeholders gather to discuss, debate and decide on policy directions. As such, they are important sites of study for illuminating dynamics in global science governance^62,63^. Our team of marine social scientists with longstanding interests in the intersections between data technologies and oceans governance used a modified Collaborative Event Ethnography (CEE) approach to engage in OOSC and UNOC3 and collectively explore our interests and observations in the proceedings. Collaborative Event Ethnography varies in the length and extent of collaboration, and in specific data collection and practices^64,65^. In this case, we relied on participant observation to structure our research collaboration. Participant observation an iterative method that involves working within a particular community to understand how it works ‘from the inside’ and documenting description of and reflection on materials and exchanges^66,67^.

To prepare for our CEE, authors held three meetings in advance of the events in which we: planned research we would present at OOSC; defined shared interests; and planned how we would observe and participate in OOSC and organize our subsequent workshop. Some authors attended OOSC and presented research; some also attended UNOC3 as accredited observers (see author attribution statement). At OOSC, authors presented research and attended sessions of interest, taking notes. Our observations are not representative of all perspectives presented at OOSC, as we designed our CEE to enable engagement in areas of the meetings reflecting team member and group interests. Following OOSC, we held a one-day workshop to discuss our observations, our experiences presenting our research, and identify themes that we were interested in pursuing collectively. Some authors also conducted a CEE at UNOC3, undertaking systematic observations of plenaries, panels, side events, and exhibitions in the diplomatic space and civil society spaces^63^. Throughout meetings and the workshop, Havice and Campbell kept detailed notes of team discussions and subsequently developed this manuscript and sought inputs from all authors.

## Data Availability

The data generated and/or analyzed during the current study are not publicly available because they consist of individuals’ notes. Clarifications are available from the corresponding author on reasonable request. Documentation of UNOC3 is available here: https://sdgs.un.org/conferences/ocean2025 and of OOSC here: https://one-ocean-science-2025.org/.
